# Daily regulation of glucocerebrosidase activity and impact of fixed-time rotarod training in a mouse model of *Gba1*-associated Parkinson's disease

**DOI:** 10.3389/fncel.2026.1924016

**Published:** 2026-10-06

**Authors:** Chiara Bertasini, Shima Tavakolian Haghighi, Maria Giusy Bruno, Fabio Paio, Carlo Alberto Artusi, Matteo Brunelli, Federico Schena, Massimo Venturelli, Ugo Moretti, Maria Grazia Romanelli, Giovanna Paolone

**Affiliations:** 1 Department of Diagnostics and Public Health, Section of Pharmacology, University of Verona, Verona, Italy; 2 Department of Neurosciences, Biomedicine and Movement Sciences, Section of Biology and Genetics, University of Verona, Verona, Italy; 3 Department of Neurosciences, Biomedicine and Movement Sciences, Section of Clinical Neurology, University of Verona, Verona, Italy; 4 Department of Diagnostics and Public Health, Section of Pathology, University of Verona, Verona, Italy; 5 Department of Neurosciences, Biomedicine and Movement Sciences, Section of Movement Sciences, University of Verona, Verona, Italy

**Keywords:** daily, fixed-time rotarod training, hSNCAA53T Gba1+/+, hSNCAA53T Gba1+/L444P, plasma GCase activity, time-of-day variation

## Abstract

**Introduction:**

Mutations in the glucocerebrosidase (*GBA1*) gene are a major genetic risk factor for Parkinson's disease (PD) and result in dysfunctional glucocerebrosidase (GCase), the lysosomal enzyme responsible for cleaving glucosylceramide into glucose and ceramide. The pathogenic *L444P* variant disrupts the enzyme structure and activity, leading to lysosomal accumulation of glucosylceramide and ultimately impairing the clearance of monomeric α-synuclein (α-syn). Given the functional crosstalk among lysosomal homeostasis, the molecular circadian clock, and physical activity, we investigated whether GCase activity exhibits daily rhythmicity, and can be modulated by daily, fixed-time rotarod activity in healthy and *Gba1*-mutant mice.

**Methods:**

C57BL/6J healthy controls and *Gba1*-mutant PD model mice were housed under a 12 h light/dark cycle, and blood samples were collected at four Zeitgeber times (ZT2, ZT8, ZT14, and ZT20), with and without daily, fixed-time rotarod training. A fluorometric assay was performed on plasma samples to investigate the daily pattern of GCase activity.

**Results:**

Our results showed that rotarod performance was influenced by genotype-dependent vulnerabilities and by daily, fixed-time physical activity, with overall better performance during the dark-phase training. The fluorometric assay revealed that GCase activity shows a ZT-dependent daily pattern in healthy mice, whereas this pattern was disrupted in *Gba1*-mutant PD models and partially rescued by timed physical activity.

**Discussion:**

Overall, both the time of day and daily fixed-time physical activity influenced GCase regulation. Our findings reveal a functional interplay between daily biological timing and lysosomal enzyme regulation, suggesting that optimizing drug dosing and timing of administration may improve the efficacy of GCase-targeted treatments.

## 1 Introduction

Parkinson's disease (PD) is the second most common neurodegenerative disease, with a global prevalence estimated at 11.7 million in 2021 (Li et al., 2025). Heterozygous mutations in the glucocerebrosidase (*GBA1*) gene represent the most prevalent genetic risk factor for PD, present in 5%−20% of patients across different populations worldwide, characterized by early onset, rapid progression, and greater cognitive decline (Marano et al., 2023, 2026; Cilia et al., 2025; Avenali et al., 2020; Riccò et al., 2020; Kucinski et al., 2013). *GBA1* encodes glucocerebrosidase (GCase), a lysosomal enzyme essential for cleaving glucosylceramide into glucose and ceramide (Vieira et al., 2024; Blanz and Saftig, 2016; Mazzulli et al., 2011). Pathogenic variants, such as the well-characterized missense mutation *L444P*, disrupt the enzyme structure and catalytic activity, leading to lysosomal accumulation of glucosylceramide and secondary global lysosomal dysfunction (Bougea, 2025; Wang et al., 2025). This decline in lysosomal function reduces the clearance of monomeric alpha-synuclein (α-syn, *SNCA* gene), promoting the accumulation and aggregation of the protein into pathogenic oligomers and fibrillar forms (Mazzulli et al., 2011). Aggregated α-syn further impairs GCase trafficking to the lysosome, reducing enzymatic activity and establishing a feedback loop that drives progressive proteostatic decline.

The h*SNCA*^A53T^ *Gba1*^+/+^and h*SNCA*^A53T^ *Gba1*^+/*L*444*P*^mutants combine GCase deficiency with α-synucleinopathy, reproducing the bidirectional pathological loop central to *GBA1*-associated PD. This model reflects the subset of PD patients carrying *GBA1* mutations characterized by substantially increased disease risk, earlier onset, and accelerated cognitive and motor decline. Phenotypically, the model recapitulates key features of early-stage disease, including impaired lysosomal function, reduced GCase activity, α-synuclein pathology, and subtle motor deficits (Migdalska-Richards et al., 2020; Tayebi et al., 2017; Fishbein et al., 2014).

Autophagic-lysosomal degradation is known to be regulated by the circadian clock, the endogenous timekeeping system that establishes 24-h rhythmicity in rest–activity patterns, feeding behavior, body temperature, and hormone secretion (Patke et al., 2020; Kuhlman et al., 2018; Paolone et al., 2012). This evidence supports the hypothesis that GCase deficiency may disrupt the rhythmic clearance processes relying on lysosomal integrity, contributing to α-syn pathology.

In parallel, emerging evidence suggests that circadian rhythm disruption may have a relevant role in the pathophysiology of PD and other neurodegenerative disorders, leading to sleep disturbances, autonomic dysfunction, depression, and altered clock gene rhythmicity (Xu et al., 2024; Yalçin et al., 2025). Together, these findings suggest that lysosomal and circadian dysfunction are interconnected contributors to PD pathophysiology rather than independent processes.

Clinical and preclinical studies suggest that physical activity serves as a powerful non-pharmacological intervention for PD, improving motor and cognitive symptoms modulating the mesolimbic dopaminergic pathway (Li et al., 2026; Marino et al., 2023; Padilha et al., 2023; Policastro et al., 2020; Sacheli et al., 2019; Hwang et al., 2018). Physical activity also acts as a chronotherapeutic factor, entraining peripheral circadian clocks via neuroendocrine signaling, collectively improving sleep quality, cognitive function, and alleviating non-motor symptoms in PD (Li et al., 2026; Duret and Nagoshi, 2025).

Moreover, exercise has been shown to activate the autophagy-lysosome system, in part through the transcriptional factor EB (TFEB)-mediated upregulation of a subset of genes involved in autophagosome formation and lysosomal biogenesis. This adaptive response is regulated by a duration-dependent manner, with greater upregulation observed after long-term compared to short-term training activity (Finkbeiner, 2020; Huang et al., 2019). Additionally, *in vivo* studies confirmed that physical activity modulates lysosomal degradation molecules and enhances α-synuclein clearance in PD mice (Jang et al., 2018). It has also been proven that peripheral and central clocks regulate the cellular physiology and coordinate the rhythmic activity of key enzymes and transporters, ensuring glycolipid homeostasis (Lin et al., 2025; Peek et al., 2015). Based on this evidence, we aimed to investigate GCase activity around the clock in mouse models of lysosomal pathology and monitor whether physical activity can modulate the pattern of activity. To this end, we employed *Gba1*- double-transgenic PD models and C57BL/6J controls housed on a 12 h light/dark cycle. We analyzed plasma GCase activity from mice blood samples collected at four zeitgeber times (ZT2, ZT8, ZT14, ZT20) during both the light and dark phases, with and without daily, fixed-time rotarod practice.

## 2 Materials and methods

### 2.1 Animals

#### 2.1.1 The transgenic mouse strain

STOCK *Gba1*^*tm*1*Rlp*^ Tg(SNCA^*^A53T)1Nbm *Snca*^*tm*1*Nbm*^ Tg(SNCA^*^A53T)2Nbm/Mmjax (RRID:MMRRC037633-JAX) was obtained from the Mutant Mouse Resource and Research Center (MMRRC) at The Jackson Laboratory, an NIH-funded repository, and a breeding colony was subsequently established and maintained at The University of Verona. The strain was originally generated by Robert L. Nussbaum and donated to the MMRRC (Fishbein et al., 2014). This line is maintained on a mixed genetic background (129S4/SvJaeJ, 129S6/SvEvTac, C57BL/6J, C57BL/6NTac, and FVB/NTac), the contribution of which cannot be fully resolved genome-wide. Both experimental lines express human A53T *alpha-synuclein* (*SNCA)* in the absence of the endogenous mouse *Snca* gene (*Snca*^−/−^) and differ only at the glucocerebrosidase (*Gba1*) locus, carrying either the wild-type allele or the L444P mutation; offspring were genotyped by PCR/sequencing (described below). Age-matched C57BL/6J mice were employed as a wild-type reference strain and maintained in an in-house breeding colony (Sarsani et al., 2019). To enable precise control of the light/dark cycle, mice were housed inside an Aria Bio-C36 Evo cabinet (Tecniplast), equipped with an innovative, dimmable LED lighting system with a daily/weekly timer. Standard laboratory conditions were maintained (22°C −24 °C, 55–65% relative humidity, 12-h light/dark cycle), with *ad libitum* access to food and water. Mice were allowed to habituate to the cabinet for two additional weeks. A total of 15 male and female mice (*n* = 5 per genotype) were analyzed at the post-symptomatic stage (16–18 months of age): C57BL/6J (three males, two females), h*SNCA*^A53T^ *Gba*1^+/+^ (two males, three females), and h*SNCA*^A53T^ *Gba*1^+/L444P^ (three males, two females). The current study was conducted in accordance with the ARRIVE (Animal Research: Reporting of *In Vivo* Experiments) guidelines. All experimental procedures involving animals complied with the guidelines of the European Directive 2010/63/EU on the protection of animals employed for scientific purposes. Experimental protocols were approved by the University of Verona Animal Welfare Committee and authorized by the Italian Ministry of Health (D.M. 246/2012-B).

### 2.2 Genotyping

Genotyping was performed by genomic PCR using DNA extracted from ear biopsy samples. Briefly, ear tissue was incubated overnight at 56 °C in 100 μL of homemade lysis buffer containing 1 μL of proteinase K [Thermo Fisher Scientific (Thermo Fisher, Waltham, MA, USA)] to ensure complete tissue digestion. Proteinase K was subsequently inactivated by heating the lysates at 90 °C, after which samples were stored at −20 °C until analysis. Before PCR amplification, lysates were diluted 1:10 in DNase- and RNase-free water. Conventional PCR was performed according to the manufacturer's instructions using custom genotype-specific primers [Thermo Fisher Scientific (Thermo Fisher, Waltham, MA, USA)]. PCR products were separated by agarose gel electrophoresis, and genotypes were determined based on the expected banding patterns.

### 2.3 Experimental plan

To investigate the interplay between lysosomal dysfunction and daily rhythmicity, as well as the modulatory effects of physical activity, plasma GCase activity was monitored around the clock in h*SNCA*^A53T^ *Gba1*^+/+^(n = 5), h*SNCA*^A53T^ *Gba1*^+/*L*444*P*^(n = 5), and C57BL/6J (n = 5) mice through serial blood draws at four Zeitgeber Times (ZT2, ZT8, ZT14, ZT20; see below; Paolone et al., 2012) in four different conditions in which mice performed the rotarod practice either during the light phase (ZT4) or dark phase (ZT16).

Because the light/dark cycle of animal facilities is typically aligned with the human active phase rather than with the mouse circadian cycle, we first collected blood when mice were housed under standard lighting conditions, with lights on at 9 AM (ZT0; *t1*) and then, after 2 weeks of daily, fixed-time rotarod activity at ZT4 (*t2*). At this point, to facilitate daily training during the mice active phase, the light/dark cycle was changed, with lights on at 9 PM (ZT0), and blood was drawn after 2 weeks of acclimation to the new light/dark cycle (*t3*) and again after 2 weeks of fixed daily rotarod activity at ZT16 (*t4*).

The timeline with major experimental procedures and endpoints is summarized in Figure 1.

**Figure 1:**
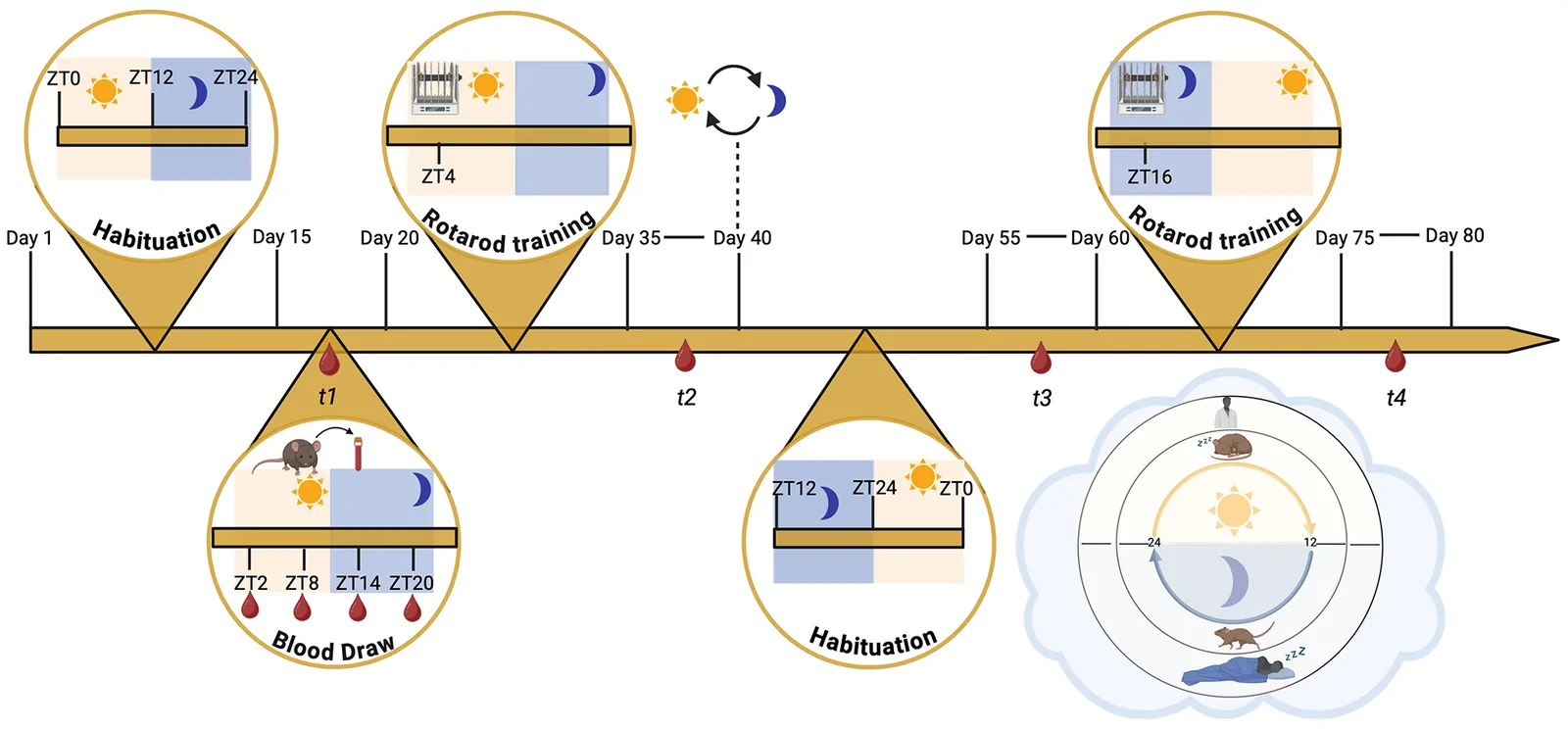
Timeline of major experimental events. Mice were housed in a standard light/dark cycle (ZT0 defined as 9:00 am, lights on; Days 1–40) and a reversed light/dark cycle (ZT0 defined as 9:00 pm, lights on; Days 41–80). Blood collection was performed at fixed ZTs (ZT2, ZT8, ZT14, ZT20) over 5 days, under standard (*t1*) and reversed (*t3*) housing conditions. Additionally, a 15-day, fixed-time rotarod training was performed at ZT4 and ZT16, each followed by blood collection (*t2* and *t4*, respectively).

### 2.4 Rotarod test

Mice underwent daily motor training and assessment using the rotarod test to evaluate motor coordination, balance, and endurance (Brooks et al., 2012; Paolone et al., 2015; Bertasini et al., 2026). Before testing, mice were habituated to the apparatus (Ugo Basile, Models 47650/47750) using an accelerating protocol in which the rod speed increased from 4 to 40 rpm over 300 s. Each testing session consisted of two consecutive trials separated by a 5-min intertrial interval. Each trial ended when the mouse fell from the rotating rod, and the latency to fall was recorded. The average latency across the two trials was calculated for each animal and used for subsequent analyses.

### 2.5 Blood collection

Blood samples were collected by submandibular venipuncture using a sterile 4-mm lancet, as previously described by Golde et al. (2005). Mice were gently, manually restrained, and whole blood was collected into 10 μL ethylenediaminetetraacetic acid (EDTA, 250 mM)-coated microcentrifuge tubes and centrifuged at 1000 × g for 10 min at 4 °C to obtain plasma, which was stored at −80 °C until analysis. To ensure mouse welfare, animals were not subjected to more than four blood draws within a 5-day interval. Blood was therefore collected once daily on four of the 5 days, with the sampling timepoint rotated across ZT2, ZT8, ZT14, and ZT20, for each experimental condition.

### 2.6 GCase assay

Plasma GCase activity was measured as previously described by Pavan et al. (2024) (Bertasini et al., 2026, in press), with minor modifications. Briefly, 5 μL of plasma was incubated with 5 μL of 2% Taurocholic acid (Sigma-Aldrich), and 50 μL of 10 mM 4-methylumbelliferyl-β-D-glucopyranoside (4-MU-β-Glc; Sigma-Aldrich) prepared in citrate-phosphate buffer (0.1 M citrate/0.2 M phosphate, pH 5.2). The reaction mixture was incubated at 37 °C for 2 h and terminated by the addition of 0.5 M carbonate buffer (pH 10.7). The fluorescence of the released 4-methylumbelliferone was measured using a microplate fluorimeter (Victor X Multilabel Plate Reader, PerkinElmer) at 365 nm excitation and 495 nm emission. GCase activity was expressed as nmol of 4-MU produced per hour per microliter of sample (nmol 4-MU/h/μL).

### 2.7 Statistical analysis

All statistical analyses were conducted using GraphPad Prism software [GraphPad Software, Inc. (GraphPad, San Diego, CA, USA) and SPSS (SPSS, Inc., Chicago, IL, USA)]. Data were analyzed using a two-way and three-way mixed-effects ANOVA with genotype as the between-subjects factor and condition (a combination of light/dark cycle and rotarod status) and ZT as the within-subjects factor, followed by Tukey's multiple-comparisons test. Mauchly's test was used to assess the assumption of sphericity. When sphericity was violated, the Geisser-Greenhouse correction was applied. Rhythmicity was further assessed by cosinor analysis, fitting plasma GCase activity to a sine wave with a fixed 24-h period using non-linear regression. For each group and condition, the fit with amplitude unconstrained was compared to a null model with amplitude fixed to zero using the extra sum-of-squares *F*-test to formally test for the presence of significant 24-h rhythmicity. To visually illustrate individual animal variability along with the group-level rhythm, we additionally fitted the linearized cosinor model (*Y* = *M* + β·cos(2π·ZT/24) + γ·sin(2π·ZT/24)) to the individual time series of each animal, and averaged the resulting parameters across animals in each genotype/condition to obtain group-level MESOR, amplitude, and acrophase estimates (Bingham et al., 1982; individual trajectories are presented in Figure 1 of the Supplementary material). A representative curve was then generated from these group-level parameters across the full 24-h cycle and plotted along with the individual raw data points. This individual-level fitting and curve generation were performed just for illustrative purposes and were not used to derive the formal statistical results, which are based on the pooled nonlinear regression and extra sum-of-squares *F*-test described above.

Importantly, cosinor and ANOVA analysis of GCase activity sometimes yielded divergent results, with ANOVA detecting significant pairwise differences between time points even when cosinor analysis found no significant 24-h rhythmicity, or vice versa. This is not unexpected, as the two tests rely on different underlying assumptions. The threshold for significance was set at *p* ≤ 0.05. Results are reported as Mean ± Standard Error of the Mean (SEM), together with sample size. Statistical results are summarized in Table 1 (rotarod test), Table 2 (GCase activity), and Supplementary Table 1 (cosinor analysis).

**Table 1:** Rotarod test.

Genotype	Main effect of time (day)	Main effect of genotype	Time × genotype interaction
*C57BL/6J*	*F*_(3,36)_ = 8.836, *p* = 0.0002	*F*_(2,12)_ = 2.865, *p = 0.0961*	*F*_(6,36)_ = 2.445, *p = 0.0437*
*SNCAA53Gba1^+/+^*	**hSNCAA53T Gba1** ^**+/+**^ *Day 20 vs. Day 60: p = 0.0020* *Day 20 vs. Day 75: p = 0.0006*		***Day 20:*** *C57BL/6J vs. hSNCAA53T Gba1^+/+^, p = 0.0101* *C57BL/6J vs. hSNCAA53T Gba1^+/*L*444*P*^, p = 0.0474*
*hSNCAA53TGba1^+/*L*444*P*^*	**hSNCAA53T Gba1** ^**+/*L*444*P***^ *Day 20 vs. 75: p = 0.0065* *Day 60 vs. 75: p = 0.0208*		***Day 60:*** *C57BL/6J vs. hSNCAA53T Gba1^+/*L*444*P*^**, p** **=** **0.0231*** *hSNCAA53T Gba1^+/+^* vs. *hSNCAA53T Gba1^+/*L*444*P*^, p* = 0.0336

Genotypes are in bold to facilitate reading.

**Table 2:** Plasma GCase activity.

Condition			ANOVA	Post-doc comparisons
**Genotype ×condition (standard housing-untrained, standard housing-trained reversed-untrained, reversed-trained) ×ZT**			**Main effect of genotype**: *F*_(2.12)_ = 46.03, *p* < 0.001 **Main effect of condition:** *F*_(3,36)_ = 28.17, *p* < 0.001 **ZT ×genotype:** *F*_(3.75,22.5)_ = 6.15, *p* = 0.002 **Condition ×ZT ×genotype:** *F*_(8.04,4.23)_ = 3.53, *p* = 0.003	**Parwise comaprisons are reported below, alligned with the order of figure presentation of the resutls**
Figure 3	**Mice maintained under standard housing condition with rotatorod training at ZT4**	**Before rotarod**	**Main effect of genotype:** *F*_(2,12)_ = 51.21, *p* < 0.001	**Before vs. after rotarod (mean, SEM)** **C57BL/6J:** before (51.40 ± 4.19), after (44.79 ± 3.64), *p* = 0.4315 **hSNCA**^**A53T**^ **Gba1**^**+/+**^**:** before (25.71 ± 1.67), after (21.52 ± 1.61), *p* = 0.0015 **hSNCA**^**A53T**^ **Gba1**^**+/L444P**^**:** before (19.75 ± 1.72), after (13.61 ± 1.53), *p* = 0.0048
		**After rotarod**	**Main effect of condition** (before vs. after rotarod) *F*_(1,12)_ = 4.9, *p* = 0.047	**Before rotarod hSNCA**^**A53T**^ **Gba1**^**+/+**^**, ZT8 vs. ZT20:** *p* = 0.0094 **hSNCA**^**A53T**^ **Gba1**^**+/L444P**^**, ZT2 vs. ZT14:** *p* = 0.0418 **ZT2**, C57BL/6J vs. hSNCA^A53T^ Gba1^+/L444P+^: *p* = 0.0487 **ZT8**, C57BL/6J vs. hSNCA^A53T^ Gba1^+/+^: *p* = 0.028; C57BL/6J vs. hSNCA^A53T^ Gba1^+/L444P^: *p* = 0.0054 **ZT14**, C57BL/6J vs. hSNCA^A53T^ Gba1^+/L444P^: *p* = 0.0173; hSNCA^A53T^ Gba1^+/+^ vs. hSNCA^A53T^ Gba1^+/L444P^: *p* = 0.003 **ZT20**, C57BL/6J vs. hSNCA^A53T^ Gba1^+/+^: *p* = 0.0126; C57BL/6J vs. hSNCA^A53T^ Gba1^+/L444P^: *p* = 0.0154 **After rotarod** C57BL/6J, ZT2 vs. ZT14: *p* = 0.0440 hSNCA^A53T^ Gba1^+/+^, ZT2 vs. ZT8: *p* = 0.0280; ZT8 vs. ZT20: *p* = 0.0307 hSNCA^A53T^ Gba1^+/L444P^, ZT2 vs. ZT20: *p* = 0.0343; ZT8 vs. ZT20: *p* = 0.0111; ZT14 vs. ZT20: *p* = 0.0484 **ZT2**, C57BL/6J vs. hSNCA^A53T^ Gba1^+/+^: *p* = 0.019; C57BL/6J vs. hSNCA^A53T^ Gba1^+/L444P^: *p* = 0.0116 **ZT8**, C57BL/6J vs. hSNCA^A53T^ Gba1^+/L444P^: *p* = 0.0156; hSNCA^A53T^ Gba1^+/+^ vs. hSNCA^A53T^ Gba1^+/L444P^: *p* = 0.0059 **ZT14**, C57BL/6J vs. hSNCA^A53T^ Gba1^+/+^: *p* = 0.026; C57BL/6J vs. hSNCAA53T Gba1+/L444P: *p* = 0.0067 **ZT20**, C57BL/6J vs. hSNCAA53T Gba1+/+: *p* = 0.0022; C57BL/6J vs. hSNCAA53T Gba1+/L444P: *p* = 0.014
Figure 4	**Mice housed under reversed light/dark cycle with rotatorod training at ZT16**	**Before rotarod**	**Main effect of genotype:** *F*_(2,12)_ = 25.12, *p* < 0.001 **Main effect of condition:** *F*_(1,12)_ = 21.17, *p* < 0.001 **Main effect of ZT:** *F*_(3,36)_ = 4.21, *p* = 0.012	**Before vs. after rotarod (mean, SEM)** **C57BL/6J:** before (64.12 ± 5.74), after (55.45 ± 4.18), *p* = 0.1544; **hSNCA**^**A53T**^ **Gba1**^**+/+**^**:** before (41.86 ± 3.25), after (24.49 ± 1.28), *p* = 0.0137; **hSNCA**^**A53T**^ **Gba1**^**+/L444P**^**:** before (39.42 ± 2.67), after (23.08 ± 1.81), *p* = 0.0151 **Before rotarod** **C57BL/6J: ZT2 vs. ZT14**, *p* = 0.0025; **ZT8 vs. ZT14**, *p* = 0.0232; **ZT14 vs. ZT20**, *p* = 0.028 **hSNCA**^**A53T**^ **Gba1**^**+/+**^, **ZT2 vs. ZT14:** *p* = 0.0188 **ZT2**, C57BL/6J vs. hSNCA^A53T^ Gba1^+/L444P^: *p* = 0.0496 **ZT14**, C57BL/6J vs. hSNCA^A53T^ Gba1^+/+^: *p* = 0.0064; hSNCA^A53T^ Gba1^+/+^ vs. hSNCA^A53T^ Gba1^+/L444P^: *p* = 0.00136
		**After rotarod**	**ZT ×genotype:** *F*_(3,36)_ = 9.37, *p* < 0.001 **Condition ×ZT ×genotype:** *F*_(3,36)_ = 2.9, *p* = 0.021	**After rotarod** **C57BL/6J: ZT2 vs. ZT14**, *p* = 0.0448; **ZT14 vs. ZT20**, *p* = 0.0036 **hSNCAA53T Gba1**^**+/+**^**, ZT8 vs. ZT20:** *p* = 0.0031; **ZT14 vs. ZT20:** *p* = 0.0117 **hSNCA**^**A53T**^ **Gba1**^**+/L444P**^**, ZT2 vs. ZT14**: *p* = 0.0183; **ZT14 vs. ZT20:** *p* = 0.0220 **ZT2**, C57BL/6J vs. hSNCA^A53T^ Gba1^+/+^: *p* = 0.0161 **ZT8**, C57BL/6J vs. hSNCA^A53T^ Gba1^+/+^: *p* = 0.0186; C57BL/6J vs. hSNCA^A53T^ Gba1^+/L444P^: *p* = 0.015 **ZT14**, C57BL/6J vs. hSNCA^A53T^ Gba1^+/+^: *p* = 0.0032; C57BL/6J vs. hSNCA^A53T^ Gba1^+/+^: *p* = 0.003 **ZT20**, C57BL/6J vs. hSNCA^A53T^ Gba1^+/+^: *p* = 0.0453; C57BL/6J vs. hSNCA^A53T^ Gba1^+/+^: *p* = 0.0167; hSNCA^A53T^Gba1^+/+^ vs. hSNCA^A53T^Gba1^+/L444P^: *p* = 0.0094

Genotypes are in bold to facilitate reading.

## 3 Results

### 3.1 Longitudinal rotarod training reveals genotype- and daily phase-dependent motor performance

Mice underwent 15 days of rotarod-based exercise training during the light phase at ZT 4 (*t2*; Figure 1), and again during the dark phase at ZT 16 (*t4*; Figure 1). In Figure 2, we summarized the retention to fall registered on the first and last days of each condition (Days 20–35 and 60–75, respectively). The ANOVA revealed a significant day × genotype interaction (*F*_(6,36)_ = 2.445, *p* = 0.0437). At the beginning of training (Day 20), both h*SNCA*^A53T^ *Gba*1^+/+^ and h*SNCA*^A53T^ *Gba*1^+/L444P^ mice spent less time on the rod than C57BL/6J controls (*p* = 0.0101 and *p* = 0.0474, respectively; Buitrago, 2004). Instead, by Day 35, all groups showed comparable performance. When the training was resumed during the dark phase (Day 60), C57BL/6J and h*SNCA*^A53T^ *Gba*1^+/+^ maintained latencies close to the maximum test duration, significantly higher compared to *Gba*1^+/L444P^ mice (*p* = 0.0231; *p* = 0.0336, respectively). By Day 75, all the groups reached the maximum test duration of 300 s. Of note, significant improvement over time was detected (*F*_(3,36)_ = 8.836, *p* = 0.0002) in h*SNCA*^A53T^ *Gba*1^+/+^ (Day 20 vs. Day 60: *p* = 0.0020; Day 20 vs. Day 75: *p* = 0.0006) and h*SNCA*^A53T^h*SNCA*^A53T^ *Gba*1^+/L444P^ mice (Day 20 vs. 75: *p* = 0.0065; Day 60 vs. 75: *p* = 0.0208). The increase in latency to fall may reflect a learning effect, as well as the impact of circadian rhythmicity on physical performance (Hesketh et al., 2026).

**Figure 2:**
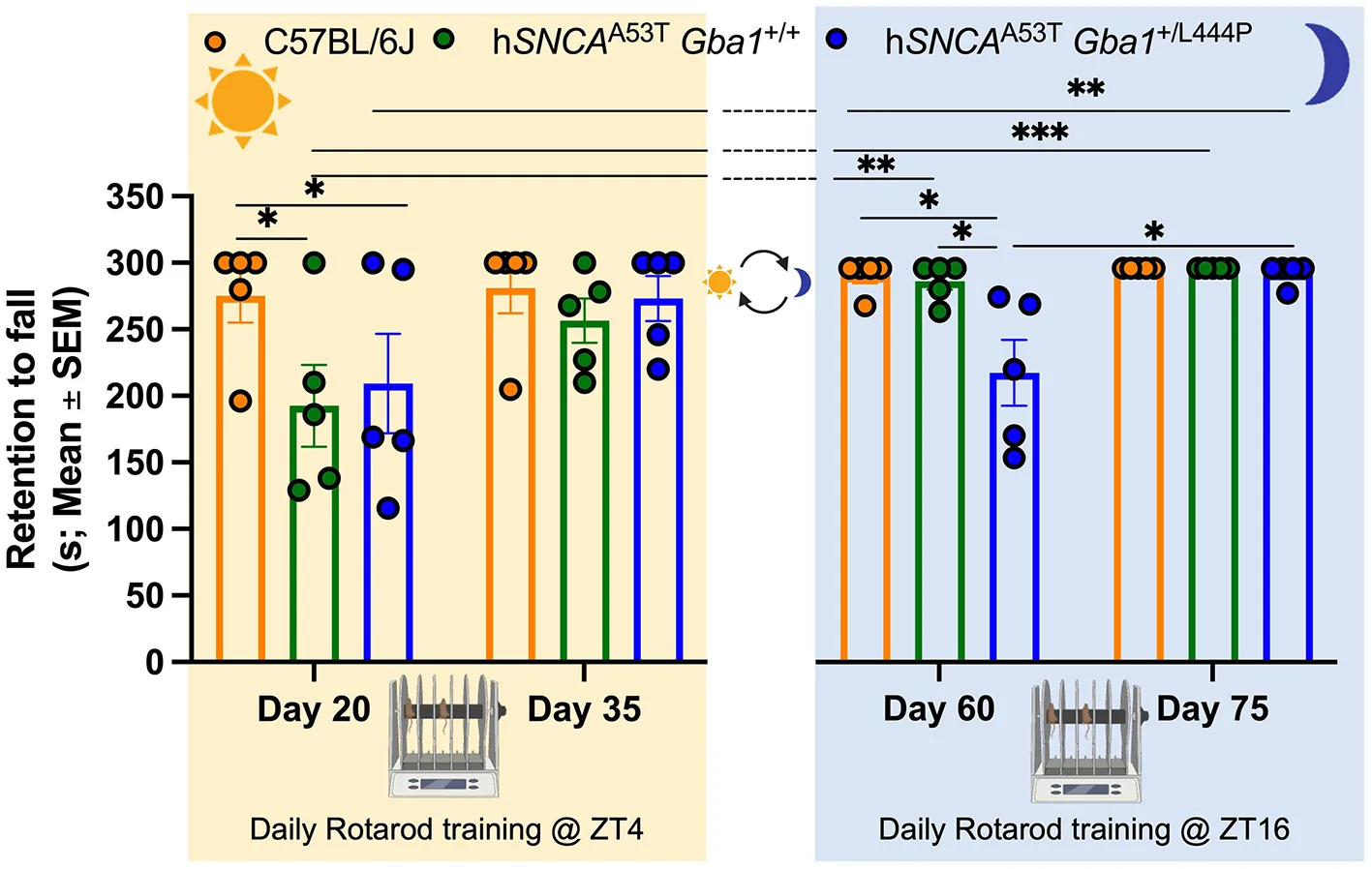
Time on the rotarod during the light and dark phases. C57BL/6J (*n* = 5), h*SNCA*^A53T^ *Gba*1^+/+^ (*n* = 5), and h*SNCA*^A53T^ *Gba*1^+/L444P^ (*n* = 5) underwent daily fixed-time rotarod training during the light phase (ZT4; Day 20–35) and during the dark phase (ZT 16; Day 60–75). Data were analyzed using two-way repeated-measures ANOVA, ^*^p < 0.05; ^**^p < 0.01; ^***^p < 0.001. On Day 20, latency to fall was lower in h*SNCA*^A53T^ *Gba*1^+/+^ and h*SNCA*^A53T^ *Gba*1^+/L444P^ mice compared with C57BL/6J controls (*p* = 0.0101 and 0.0474, respectively), whereas no genotype differences were observed on Day 35. On Day 60, h*SNCA*^A53T^ *Gba*1^+/L444P^ mice showed lower latency than C57BL/6J and h*SNCA*^A53T^ *Gba*1^+/+^ mice (*p* = 0.0231 and 0.0336, respectively). By Day 75, all groups reached the maximum test duration of 300 s. Significant within-genotype improvements were detected in h*SNCA*^A53T^ *Gba*1^+/+^ mice (Day 20 vs. Day 60, *p* = 0.0020; Day 20 vs. Day 75, *p* = 0.0006) and h*SNCA*^A53T^ *Gba*1^+/L444P^ mice (Day 20 vs. Day 75, *p* = 0.0065; Day 60 vs. Day 75, *p* = 0.0208).

### 3.2 Daily pattern of GCase activity and effects of fixed-time rotarod training

Growing evidence indicates that lysosomal and autophagic function is under circadian control, with core clock components such as BMAL1 and TFEB driving rhythmic oscillations in lysosomal activity (McKee et al., 2023). We therefore assessed whether the light/dark cycle (standard housing and reversed) and physical exercise modulate GCase activity by measuring plasma enzymatic activity at four ZT times across the 24-h cycle (ZT2, ZT8, ZT14, and ZT20) in healthy control C57BL/6J and PD-mutant mice, prior to and following 2 weeks of daily, fixed-time rotarod training performed during the light (ZT4) and dark phase (ZT16). Figure 1 depicts the major experimental events.

Data were analyzed using the general linear model (GLM) repeated measures ANOVA, with genotype as the between-subjects factor and two within-subjects factors: condition (a combination of light/dark cycle and rotarod status: standard housing-untrained, standard housing-trained; reversed-untrained, reversed-trained) and the ZT (ZT2, ZT8, ZT14, ZT20). Although this statistical model was applied to the full dataset, to aid interpretation of the phase-specific effects, results are presented with the standard and reversed light/dark housing conditions shown separately: Figure 3 shows GCase activity in untrained (A, C) and trained (B, D) animals during the light (inactive) phase under standard housing conditions, while Figure 4 shows GCase activity in untrained (A, C) and trained (B, D) animals during the dark (active) phase under reversed light/dark conditions.

**Figure 3:**
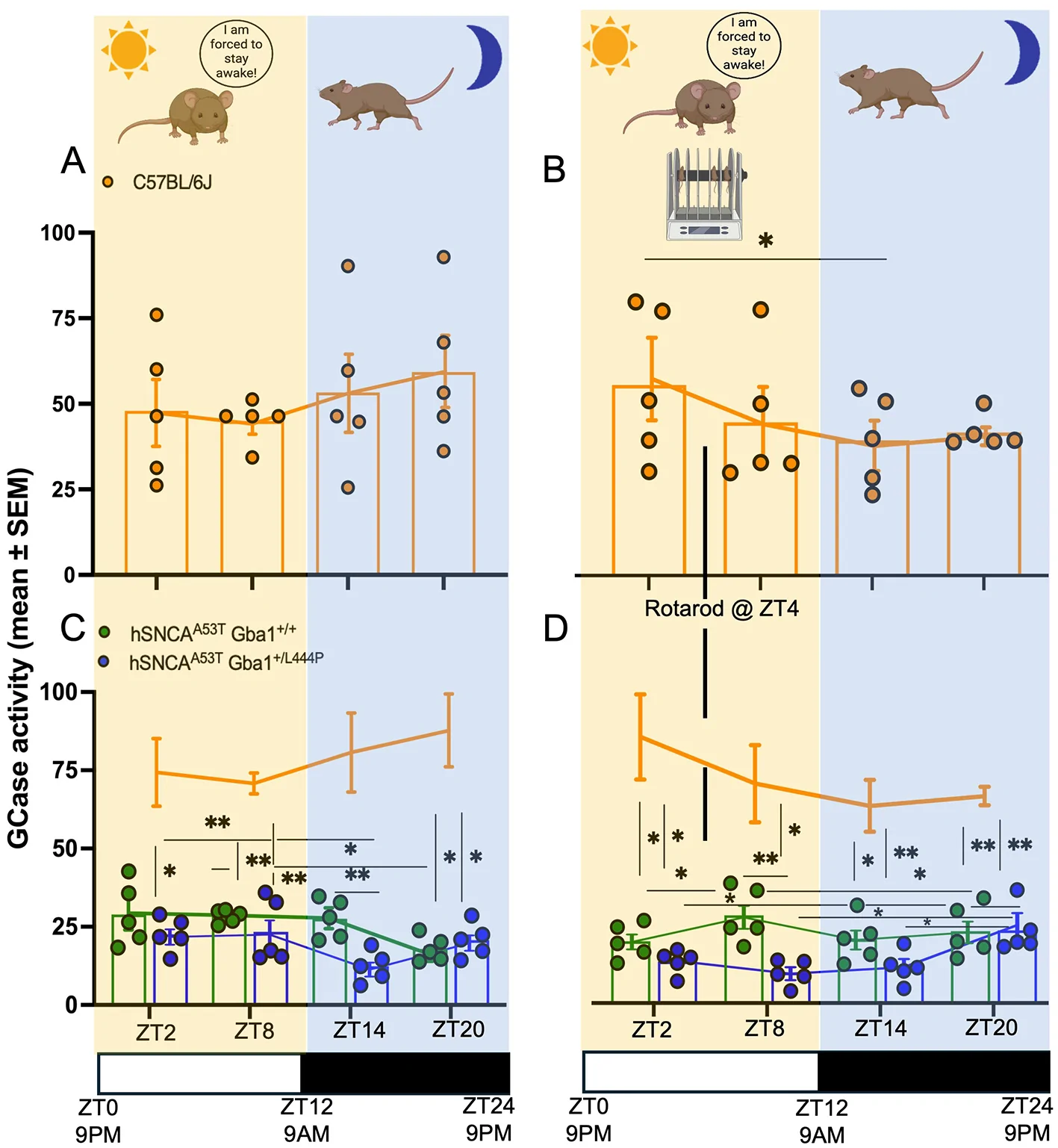
GCase activity and effects of daily, fixed-time rotarod training performed during the light (resting) phase. Plasma GCase activity at ZT2, ZT8, ZT14, ZT20, of C57BL/6J control (*n* = 5; A-B), h*SNCA*^A53T^ *Gba*1^+/+^ and h*SNCA*^A53T^ *Gba*1^+/L444P^ (*n* = 5; C–D) mice housed under standard housing conditions with light/dark cycles (ZT0 defined as = 9 AM, lights on). Measurements were performed before **(A, C)** and after **(B, D)** 15 days of daily fixed-time rotarod training at ZT4. Data were analyzed by two-way repeated-measures ANOVA **(A–D)**. Significance defined as *p < 0.05; **p < 0.01. Before training **(C)**, within-genotype differences were detected between ZT8 and ZT20 in h*SNCA*^A53T^ *Gba*1^+/+^ (*p* = 0.0094), and between ZT2 and ZT14 in h*SNCA*^A53T^ *Gba*1^+/L444P^ (*p* = 0.0418), while between genotypes statistics showed C57BL/6J differed from h*SNCA*^A53T^ *Gba*1^+/+^ at ZT8 (*p* = 0.0028) and ZT20 (*p* = 0.0126), and from h*SNCA*^A53T^ *Gba*1^+/L444P^ at ZT2, ZT8, ZT14, and ZT20 (*p* = 0.0487, 0.0054, 0.0173, and 0.0154, respectively), and significant differences were detected between h*SNCA*^A53T^ *Gba*1^+/+^ and h*SNCA*^A53T^ *Gba*1^+/L444P^ mice at ZT14 (*p* = 0.0030). After training **(B, D)**, significant within-genotype differences were found in C57BL/6J (ZT2 vs. ZT14, *p* = 0.044), h*SNCA*^A53T^ *Gba*1^+/+^ (ZT2 vs. ZT8, *p* = 0.028; ZT8 vs. ZT20, *p* = 0.0307), and h*SNCA*^A53T^ *Gba*1^+/L444P^ mice (ZT2 vs. ZT20, *p* = 0.0343; ZT8 vs. ZT20, *p* = 0.0111; ZT14 vs. ZT20, *p* = 0.0484). Moreover, C57BL/6J differed from h*SNCA*^A53T^ *Gba*1^+/+^ at ZT2, ZT14, and ZT20 (*p* = 0.019, 0.026, and 0.0022), and from h*SNCA*^A53T^ *Gba*1^+/L444P^ at all four time points (*p* = 0.0116, 0.0156, 0.0067, and 0.0014 for ZT2, ZT8, ZT14, and ZT20, respectively), and significant differences were detected between h*SNCA*^A53T^ *Gba*1^+/+^ and h*SNCA*^A53T^ *Gba*1^+/L444P^ mice at ZT8 (*p* = 0.0059).

**Figure 4:**
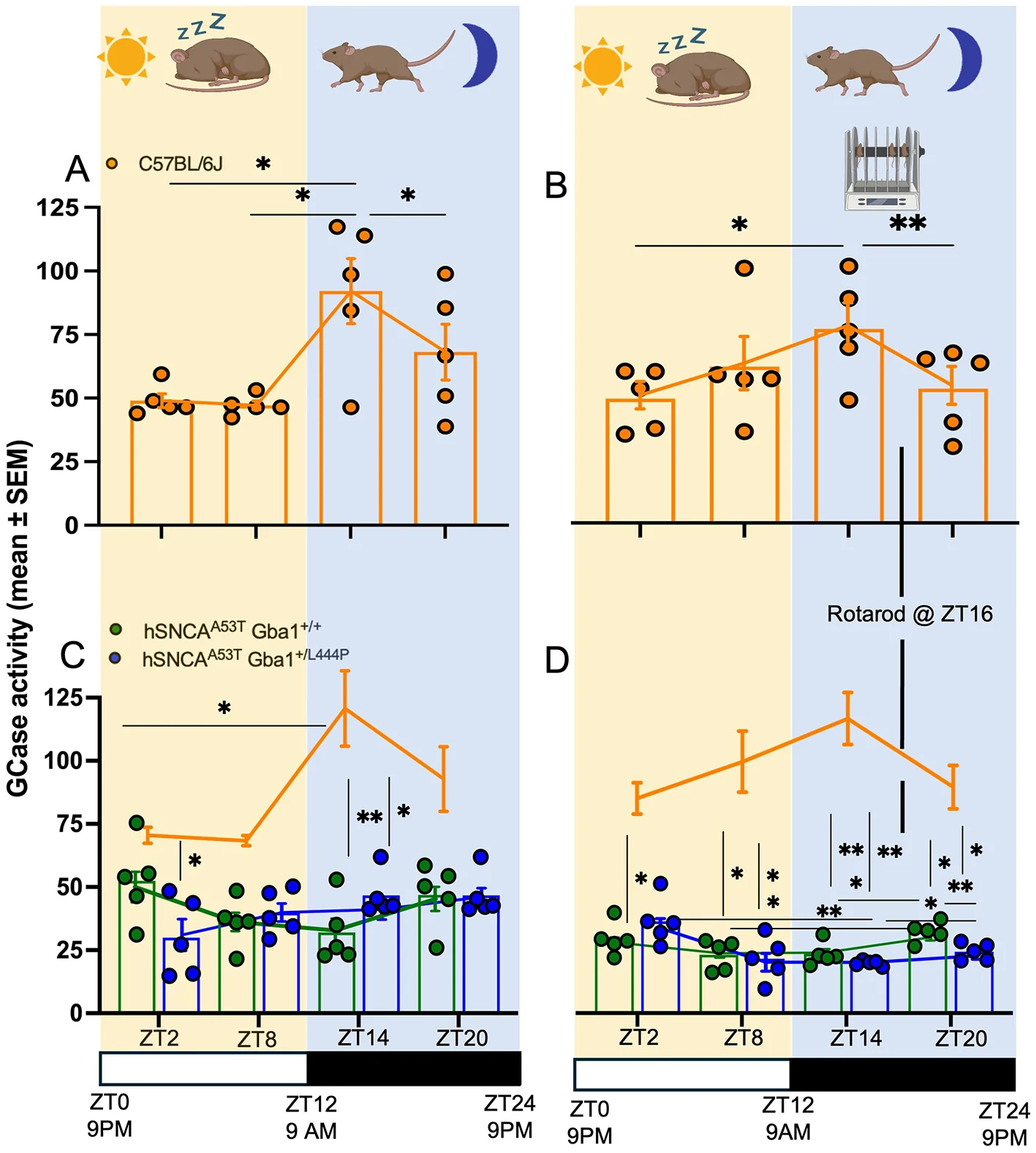
GCase activity and effects of fixed-time rotarod training during the dark (active) phase. Plasma GCase activity was measured at ZT2, ZT8, ZT14, and ZT20 in C57BL/6J **(A, B)**, h*SNCA*^A53T^ *Gba*1^+/+^, and h*SNCA*^A53T^ *Gba*1^+/L444P^ mice (C-D; *n* = 5/genotype) housed under a reversed light/dark cycle (ZT0 = 9PM, lights on). GCase activity is shown before **(A, C)** and after **(B, D)** 15 days of daily rotarod training performed at ZT16. Data were analyzed by two-way repeated-measures ANOVA. Significance defined as *p < 0.05; **p < 0.01; ***p < 0.001. Before training, significant within-genotype differences were observed in C57BL/6J mice between ZT2 and ZT14 (*p* = 0.0254), ZT8 and ZT14 (*p* = 0.0232), and ZT14 and ZT20 (*p* = 0.028), and in h*SNCA*^A53T^ *Gba*1^+/+^ mice between ZT2 and ZT14 (*p* = 0.0188). Between-genotype differences were detected at ZT14 between C57BL/6J and h*SNCA*^A53T^ *Gba*1^+/+^ (*p* = 0.0064), and between C57BL/6J and h*SNCA*^A53T^ *Gba*1^+/L444P^ mice at ZT2 (*p* = 0.0496), and ZT14 (*p* = 0.0136). After training, significant within-genotype differences were observed in C57BL/6J between ZT2 and ZT14 (*p* = 0.0448) and ZT14 and ZT20 (*p* = 0.0036); in h*SNCA*^A53T^ *Gba*1^+/+^ between ZT8 and ZT20 (*p* = 0.0031) and ZT14 and ZT20 (*p* = 0.0117); and in h*SNCA*^A53T^ *Gba*1^+/L444P^ between ZT2 and ZT14 (*p* = 0.0183), and Z14 and ZT20 (*p* = 0.022). Genotype comparison revealed differences between C57BL/6J and h*SNCA*^A53T^ *Gba*1^+/+^ mice at ZT2, ZT8, ZT14, and ZT20 (*p* = 0.0161, 0.0186, 0.0032, and 0.0453, respectively), and between C57BL/6J and h*SNCA*^A53T^ *Gba*1^+/L444P^ mice at ZT8, ZT14, and ZT20 (*p* = 0.015, 0.003, and 0.0167, respectively), and significant differences were detected between h*SNCA*^A53T^ *Gba*1^+/+^ and h*SNCA*^A53T^ *Gba*1^+/L444P^ mice at ZT20 (*p* = 0.0094).

The omnibus GLM revealed a significant main effect of genotype *F*_(2,12)_ = 46.03, *p* < 0.0001, a significant main effect of condition *F*_(3,36)_ = 28.17, *p* < 0.0001, a significant genotype × ZT *F*_(3.75,22.5)_ = 6.15, *p* = 0.002 and genotype × condition × ZT *F*_(8.04,48.23)_ = 3.53, *p* = 0.003 interaction indicating that bot circadian time and rotarod training modulate GCase activity in a genotype-dependent manner. These effects are examined in detail in the sections below and summarized in Table 2.

#### 3.2.1 GCase activity and effects of daily, fixed-time rotarod training performed during the light (resting) phase of mice maintained under standard housing conditions

We first assessed the impact of standard housing conditions (with ZT0 defined as 9:00 AM, lights on; Hawkins and Golledge, 2018) on GCase activity and evaluated whether daily, fixed-time rotarod training at ZT4 further modulates the activity pattern.

To further characterize the significant genotype × condition × ZT interaction, follow-up repeated measure analyses (genotype as the between-subjects factor; training and ZT, as within-subject factors) were performed for the standard and reversed housing conditions.

GCase activity differed significantly by genotype (*F*_(2,12)_ = 51.21, *p* < 0.001). Rotarod training had a modest but significant effect on GCase activity, (*F*_(1,12)_ = 4.9, *p* = 0.047) and this effect do not depend on genotype (training × genotype, (*F*_(2,12)_ = 0.08, *p* = 0.920). Unlike the reversed cycle condition (below), GCase activity did not vary significantly across ZT during the standard housing condition (*F*_(3,36)_ = 1.28, *p* = 0.295) nor was the ZT × genotype interaction significant, (*F*_(6,36)_ = 1.98, *p* = 0.094). Similarly, neither the training by ZT interaction, (*F*_(3,36)_ = 0.14, *p* = 0.935), nor the training × ZT × genotype interaction, (*F*_(6,36)_ = 1.78, *p* = 0.131), reached significance.

In healthy control C57BL/6J mice (Figure 3A), GCase activity showed an increasing trend during the dark phase, although this did not reach any statistical significance, while the pattern in h*SNCA*^A53T^ *Gba*1^+/+^ mice (Figure 3C), was characterized by relatively stable activity with a significant decrease at ZT20 compared to ZT8 (*p* = 0.0094). The difference compared to control was significant at ZT8 (p = 0.0028), and ZT20 (*p* = 0.0126). Additionally, in h*SNCA*^A53T^ *Gba*1^+/L444P^ mice the within-group changes reached significance at ZT14 relative to ZT2 (*p* = 0.0418), while significant differences relative to the control emerged at ZT2 (*p* = 0.0487), ZT8 (*p* = 0.0054), ZT14 (*p* = 0.0173), and ZT20 (*p* = 0.0154). Furthermore, significant differences were detected between h*SNCA*^A53T^ *Gba*1^+/+^ and h*SNCA*^A53T^ *Gba*1^+/L444P^ mice at ZT14 (*p* = 0.0030).

Daily training at ZT4 affected the pattern of the enzyme activity of C57BL/6J mice, with activity slightly increased at ZT2 and progressively decreasing through ZT14 (ZT2 vs. ZT 14, *p* = 0.0440), compared to untrained. The profile also changed in h*SNCA*^A53T^ *Gba*1^+/+^ mice (Figure 3D), with a reduction in activity at ZT2, leading to significant differences between ZT2 and ZT8 (*p* = 0.0280), as well as between ZT8 and ZT20 (*p* = 0.0307). Additionally, significant differences relative to trained C57BL/6J mice were reported at ZT2 (*p* = 0.0190), ZT14 (*p* = 0.0260) and ZT20 (*p* = 0.0022). Similarly, daily training at ZT4 (Figure 3D), reduced the pattern of the enzyme activity during the light phase in h*SNCA*^A53T^ *Gba*1^+/L444P^ mice, leading to significant differences between all the ZTs and ZT20 (ZT2 vs. ZT20: *p* = 0.0343; ZT8 vs. ZT20: *p* = 0.0111; ZT14 vs. ZT20: *p* = 0.0484). Moreover, significant differences relative to trained C57BL/6J were reported at ZT2 (*p* = 0.0116), ZT8 (*p* = 0.0156), ZT14 (*p* = 0.0067) and ZT20 (*p* = 0.0014). As well as significant differences were detected between h*SNCA*^A53T^ *Gba*1^+/+^ and h*SNCA*^A53T^ *Gba*1^+/L444P^ mice at ZT8 (*p* = 0.0059).

Collectively, these data indicate that, under standard housing conditions, GCase pattern of activity is altered and is differentially modulated by motor training across genotypes, with both PD-mutant genotypes displaying, as expected, consistently reduced activity compared to controls, regardless of the training status.

#### 3.2.2 GCase activity and effects of daily, fixed-time rotarod training performed during the dark (active) phase of mice housed under a reverse light/dark conditions

We next assessed the impact of reverse-cycle housing, with ZT0 (lights on) set at 9:00 pm, allowing testing during mice active (dark) phase within standard working hours (Hawkins and Golledge, 2018).

According to the follow-up repeated measure analyses (genotype as the between-subjects factor; training and ZT, as within-subject factors) performed for reversed housing conditions, GCase activity differed significantly by genotype, *F*_(2,12)_ = 25.12, *p* < 0.001. Rotarod training significantly affected GCase activity *F*_(1,12)_ = 31.17, *p* < 0.001 and this effect did not depend on genotype (training × genotype: *F*_(2,12)_ = 1.18, *p* = 0.342. GCase activity also varied across ZT, *F*_(3,36)_ = 4.21, *p* = 0.012, and this pattern differed by genotype as indicated by a significant ZT × genotype interaction, *F*_(6,36)_ = 9.37, *p* < 0.001. The training × ZT interaction was not significant, *F*_(3,36)_ = 2.26, *p* = 0.98 but the training × ZT × genotype was significant, *F*_(6,36)_ = 2.89, *p* = 0.021, indicating that the combined effect of training and time of the day on GCase activity differed across genotype.

In untrained C57BL/6J control mice (Figure 4A), GCase activity reached significance at ZT2 vs. ZT14 (*p* = 0.00254); ZT8 vs. ZT14 (*p* = 0.0232), and ZT14 vs. ZT20 (*p* = 0.028). Rather than peaking during the dark phase as in C57BL/6J mice, GCase activity of h*SNCA*^A53T^ *Gba*1^+/+^ mice was higher at ZT2, reaching significant differences when compared to the activity at ZT14 (*p* = 0.0188; Figure 4C). Additionally, a significant difference was observed between control and h*SNCA*^A53T^ *Gba*1^+/+^ mice in the activity assessed at ZT14 (*p* = 0.0064).

As expected, also untrained h*SNCA*^A53T^ *Gba*1^+/L444P^ mice (Figure 4C) showed a pattern of GCase activity that was strongly reduced compared to control and flat around the clock, further highlighting the impact of *Gba*1^+/L444P^ genetic background on enzyme activity. Significant differences were observed between control and h*SNCA*^A53T^ *Gba*1^+/L444P^ at ZT2 (*p* = 0.0496) and at ZT14 (*p* = 0.00136 Figure 3C), while no significant differences were found when compared to h*SNCA*^A53T^ *Gba*1^+/+^ mice.

Following daily rotarod training (Figure 4B), the within-group changes of C57BL/6J control mice reached significance at ZT2 vs. ZT14 (*p* = 0.0448) and ZT14 vs. ZT20 (*p* = 0.0036).

Rotarod training further modified the pattern of GCase activity in *hSNCA*^*A*53*T*^ *Gba1*^+/+^ mice (Figure 4D), reaching significance at ZT8 vs. ZT20 (*p* = 0.0031), as well as ZT14 vs. ZT20 (*p* = 0.0117). Although the enzyme showed a more defined temporal pattern of activity following daily exercise, overall levels remained significantly lower relative to controls at each ZT (ZT2: *p* = 0.0161, ZT8: *p* = 0.0186, ZT14: *p* = 0.0032, and ZT20: *p* = 0.0453).

After daily, fixed-time rotarod training, although the overall GCase activity of h*SNCA*^A53T^ *Gba*1^+/L444P^ mice remained low, the activity pattern become rearranged, resulting in significant differences at ZT2 vs. ZT14 (*p* = 0.0183), Z14 vs. ZT20 (*p* = 0.0220) and, compared to C57BL/6J mice, at ZT8 (*p* = 0.0150), ZT14 (*p* =0.0030) and ZT20 (*p* = 0.0167). Furthermore, significant differences were detected between h*SNCA*^A53T^ *Gba*1^+/+^ and h*SNCA*^A53T^ *Gba*1^+/L444P^ mice at ZT20 (*p* = 0.0094).

Overall, as expected, genotype consistently affected GCase activity across both housing conditions. Rotarod training also significantly modulated GCase activity in both housing conditions, although this effect was more robust when training occurred during mice active phase. Interestingly, GCase activity itself varied with time of the day, as reflected in significant ZT and ZT × genotype effects, an observation that, to our knowledge, has not been previously described.

## 4 Discussion

*GBA1* mutations represent the most prevalent genetic risk factor for PD and are associated with lysosomal dysfunction, circadian abnormalities, and impaired motor and non-motor performance. Moreover, bidirectional interactions exist between circadian rhythms and lysosomal homeostasis, and emerging evidence suggests that physical activity may modulate these interactions. As a result, these molecular insights may be instrumental in identifying potential circadian, exercise-modulated therapeutic strategies to alleviate motor and non-motor symptoms of PD (Li et al., 2026; Bougea, 2025; Marano et al., 2023; Wahlberg et al., 2020). Building on this, we investigated the pattern of plasma GCase activity in mouse models of lysosomal pathology and healthy controls at four zeitgeber times, during both the active and inactive phases, with and without daily, fixed-time rotarod practice.

We first assessed the pattern of GCase activity in healthy controls under baseline conditions, and then challenged the system to monitor the impact of changes in the light/dark cycle with or without fixed-time physical activity. Rotarod performance was affected by both genotype-dependent vulnerabilities and daily, fixed-time physical activity. At baseline (Day 20, prior to the onset of training), both mutant mouse models exhibited significantly shorter retention to fall compared to healthy controls, consistent with genotype-associated motor impairments. Following 2 weeks of daily, fixed-time activity, rotarod retention times in the mutant mice increased to levels comparable to those of controls (Day 35). However, when the light/dark cycle was inverted, and the daily training was interrupted for 2 weeks, the retention to fall of h*SNCA*^A53T^ *Gba*1^+/L444P^ mice returned to levels significantly lower than both controls and h*SNCA*^A53T^ *Gba*1^+/+^ (Day 60), likely reflecting greater motor deficits present in this model (Lado et al., 2025). Two weeks of training performed in the mice active phase were sufficient to rescue performance to wild-type control levels (Day 75), suggesting that motor learning remained intact and that properly timed exercise can restore performance even in the presence of genetic vulnerability. These findings align with previous reports indicating that 4 weeks of rotarod training rescued motor deficits and reduced nigrostriatal dopaminergic degeneration in MPTP-treated mice (Leem et al., 2022), and that intensive treadmill training improved motor deficits via enhanced synaptic plasticity in an α-synuclein preformed fibril model (Marino et al., 2023). Exercise has also been associated with region-specific transcriptional changes, further supporting physical activity-induced neuroplasticity (Tong et al., 2022), as well as with reduced oxidative stress and inflammation, and increased expression of neurotrophic factors (Palasz et al., 2019). We also observed that both PD mutants performed significantly better during their active phase than during the shifted phase, suggesting that aligned scheduled exercise with the endogenous activity phase improves motor performance, consistent with evidence that endurance performance in mice varies with time of day and that exercise timing influences training adaptation, potentially also through circadian regulation of skeletal muscle oxidative metabolism (Hesketh et al., 2026; Liu et al., 2025).

To investigate the daily pattern of GCase activity, we performed a fluorometric assay on plasma samples collected at four zeitgeber times, during the active and inactive phases, prior to and after 2 weeks of rotarod training. The results revealed significant changes between light and dark phase in GCase activity in C57BL/6J animals, with activity remaining stable during the rest phase and peaking in the early active phase. Following 2 weeks of timed physical activity at ZT16, the overall profile remained, but the ZT14 peak became less pronounced, and GCase activity showed a more uniform distribution across the day, suggesting that the enzyme activity is regulated by the biological clock and is modified when combined with a scheduled physical activity. As expected, the activity of GCase was disrupted in both PD-mutant models. In the h*SNCA*^A53T^ *Gba*1^+/+^ model, this pattern was inverted relative to healthy mice: enzyme activity was significantly higher during the rest/inactive phase and declined during the early active phase, suggesting that α-synuclein overexpression reduces GCase activity and disrupts time-of-day regulation. In contrast, the h*SNCA* ^A53T^ *Gba*1^+/L444P^ mice showed a comparable reduced overall activity with an essentially flat activity pattern, with no statistically significant variation across the four ZTs.

Two weeks of physical practice, partially reorganized GCase in both mutant PD mice, although overall levels remained lower compared to the C57BJ/6L mice. In both models, exercise produced a more defined temporal profile: h*SNCA*^A53T^ *Gba*1^+/+^ showed higher activity during the late active phase (ZT20) with reduced within-group variability, whereas h*SNCA* ^A53T^ *Gba*1^+/L444P^ mice showed significantly higher activity at the onset of the resting phase relative to other ZTs. These findings align with previous studies showing that exercise can modulate lysosomal biogenesis and autophagy-related pathways through AMP-activated protein kinase-silent information regulator 1-TFEB (AMPK-SIRT1-TFEB) signaling. In α-synuclein models specifically, treadmill exercise reduces α-syn spreading and protects dopaminergic neurons via mechanisms dependent on PPARα, a nuclear receptor and transcription factor regulating genes involved in lipid metabolism, energy handling, and lysosomal/autophagy pathways. Given the established interaction between α-synuclein and GCase, and the fact that *GBA1* mutations impair GCase activity and lysosomal function, our findings suggest that scheduled physical activity may not only modulate total enzyme activity but also the pattern of activity across the day (Marano et al., 2023; Li et al., 2026; Hwang et al., 2018).

This pattern of GCase activity was lost when animals were maintained under standard housing conditions, whereas daily, fixed physical activity partially reversed the trend. These findings suggest that biological clocks and physical activity may act in concert: as exercise itself represents a robust zeitgeber, it may contribute reinforce daily rhythms across genotypes, thereby supporting GCase activity. This is also consistent with evidence that peripheral clocks (e.g., muscle; Kemler et al., 2020) are themselves entrained by physical activity.

Consistently, GCase activity was reduced in both PD mutant models, with a similarly altered pattern: activity was almost flatten, with the lowest point occurring in the active phase, a profile substantially different from the one observed under reversed light/dark condition. Following 2 weeks of rotarod practice, both genotypes showed partial reorganization of the GCase activity pattern, although neither showed full recovery of overall enzyme levels: activity in h*SNCA*^A53T^ *Gba*1^+/+^ mice peaked in the late resting phase, immediately after training, suggesting a partial exercise effect, while in h*SNCA*^A53T^ *Gba*1^+/L444P^ mice, activity shifted significantly higher during the late active phase.

Some limitations should be reported. C57BL/6J mice were used as a wild-type reference strain rather than a background-matched control, as the genetic quality control of the transgenic line (MMRRC RRID:MMRRC_037633-JAX) detected diagnostic alleles consistent with partial C57BL/6 ancestry alongside the predominant 129 and FVB/N contributions, indicating that this strain choice, while not fully background-matched, is not entirely unrelated to the genetic composition of the transgenic line (Sarsani et al., 2019). The relatively small sample size (*n* = 5/genotype), though consistent with the longitudinal repeated-measures design and the 3Rs principle of reduction, limits statistical power. Lighting schedule, exercise timing, and handling schedule were also varied together across experimental conditions, limiting the ability to dissociate their relative contribution to the observed changes in GCase activity, although this co-variation is arguably closer to real-world circadian disruption, such as that seen in jet lag or in neurological and psychiatric patients. Entrainment to the reversed light/dark cycle was not independently verified before starting the exercise protocol, a 2-week acclimation period was applied, consistent with established practice (Munn et al., 2011), but actographic assessment would have directly confirmed stable re-entrainment. All experiments were performed under light/dark cycles, without testing under constant conditions (e.g., constant lights or darkness; Paolone et al., 2012) or direct manipulation of the molecular clock. Our data, therefore, reflect ZT-dependent daily variation in GCase activity rather than formally demonstrated endogenous circadian regulation; future studies should address this directly. Moreover, GCase activity was measured in plasma, a clinically accessible, minimally invasive readout that may not directly reflect CNS lysosomal enzyme activity, therefore, the implications discussed here for brain lysosomal function and PD pathology should be considered indicative rather than directly demonstrated. Finally, we acknowledge that measuring GCase protein and/or mRNA levels would provide mechanistic insight into whether exercise at ZT4 or ZT16 triggers a specific molecular cascade involved in the reduced activity of the enzyme observed after training in each genotype.

Additionally, the cosinor analysis provided in the supplementary section, presents its own limitations. Because each animal contributed repeated measurements across the four sampling time points, the group-level cosinor fits reflect population-average rhythmic patterns rather than independently sampled observations. While individual-animal cosinor fitting would, in principle, better respect this repeated-measures structure, four time points per 24-h cycle provide limited degrees of freedom for reliably estimating individual rhythm parameters (e.g., mesor, amplitude, and acrophase). This sampling density was constrained by the need to repeatedly sample the same animals across four experimental conditions through the submandibular venipuncture, to our knowledge, the least invasive technique available, while remaining compatible with animal welfare considerations and the 3Rs principles. In addition, a repeated-measures ANOVA, which appropriately accounts for the non-independence of repeated sampling within each animal, confirmed a significant effect of time of day on plasma GCase activity. This result, together with the consistency of individual animal trajectories shown in Supplementary Figure 1, supports the conclusion that the rhythmic pattern identified by cosinor analysis reflects a genuine biological phenomenon rather than a statistical artifact of the analytical approach.

These findings may provide relevant therapeutic implications for *Gba1*-associated PD. Recognizing that GCase activity follows a ZT-dependent pattern of activity and is modulated by exercise suggests that timing-based approaches, optimizing drug dosing and administration timing, could improve the efficacy of GCase -targeted treatments (West et al., 2017; Kudo et al., 2011).

## 5 Conclusion

Our findings indicate a close functional interaction between lysosomal homeostasis, the molecular clock, and physical activity. Rotarod performance data suggest that aligning fixed-time physical activity with the endogenous activity rhythm improves performance in *Gba1*-mutant PD models. We demonstrated that plasma GCase activity follows a ZT-dependent variation in healthy control mice, and that this pattern is significantly disrupted in *Gba1*-mutant PD models. Daily timed physical activity partially restored enzyme activity when performed during the mice active phase. Importantly, both exercise and daily timing modulated GCase activity. These findings may provide important insights into the molecular mechanisms underlying impaired GCase pattern of activity, ultimately leading to lysosomal glucosylceramide accumulation, impaired clearance of α-syn, and progressive neurodegeneration in *GBA1*-associated PD. Future studies should monitor level GCase protein and/or mRNA levels and prioritize optimizing treatment timing in GCase-targeted therapies for *GBA1*-associated PD, with exercise scheduling as a potential adjusting strategy.

## Data Availability

The original contributions presented in the study are included in the article/Supplementary material, further inquiries can be directed to the corresponding author.
