# Supplementary material for: Daily regulation of glucocerebrosidase activity and impact of fixed-time rotarod training in a mouse model of Gba1-associated Parkinson's disease
Source: Front Cell Neurosci. 2026 Oct 6;20:1924016. doi: 10.3389/fncel.2026.1924016 (PMC13680190; doi:10.3389/fncel.2026.1924016)
Supplement: Supplementary file 1 [file Table_4.docx]

Supplementary Material

# 1 Supplementary Data

*Cosinor analysis of plasma GCase activity across genotypes, housing conditions, and training status*

To provide a descriptive characterization of the temporal pattern of GCase activity, cosinor curves were additionally fitted to the pooled data using nonlinear regression (Supplementary Figure 1). As these fits do not account for the repeated-measures structure of the data (four time points per animal), the resulting F statistics and P values should be interpreted as descriptive indices of goodness of fit rather than as formal, independent confirmation of rhythmicity; primary statistical inference on time-of-day and group effects is based on the repeated-measures ANOVA reported in the main text, summarized in table 2, and presented in Figure 3 and Figure 4.

When mice were housed under standard housing conditions (*t1*), cosinor analysis did not detect significant 24-hour rhythmicity in C57BL/6J (Supplementary Figure 1A; F_(1,17)_ = 1.639, p = 0.2177; amplitude = 7.665, 95% CI 4.968-20.30; R² = 0.088). In contrast, cosinor analyses reached significance in both h*SNCA*^A53T^ *Gba1*^+/+^ mice (Supplementary Figure 1B; F_(1,17)_ = 6.053, p = 0.0249; amplitude = 5.272, 95% CI 0.751-9.792; R² = 0.263) and h*SNCA*^A53T^ *Gba1*^+/L444P^ mice (Supplementary Figure 1C; F_(1,17)_ = 5.653, p = 0.0294; amplitude = 5.310, 95% CI 0.598-10.02; R² = 0.250). After two weeks of daily training during the light (inactive) phase at ZT4 (*t2*), this pattern was modified, however, cosinor analysis performed on GCase activity of C57BL/6J mice showed not significant rhythmicity (Supplementary Figure 1D; F_(1,17)_ = 2.523, p = 0.1306; amplitude = 8.055, 95% CI 2.645-18.76; R² = 0.129). Similarly, in h*SNCA*^A53T^ *Gba1*^+/+^ mice, no significant 24-hour rhythmicity was detected (Supplementary Figure 1E; F_(1,17)_ = 1.066, p = 0.3163; amplitude = 2.418,95% CI 3.981-7.359, R² = 0.059). By contrast, training strengthened the pattern of GCase activity in h*SNCA*^A53T^ *Gba1*^+/L444P^ mice, with the cosinor analysis reaching significance (Supplementary Figure 1F; F_(1,17)_ = 14.74, p = 0.0013; amplitude = 6.427, 95% CI 2.895-9.959; R² = 0.464).

*Please, insert Supplementary Table 1 about here.*

Under reversed light/dark conditions (*t3*), cosinor analysis was consistent with significant 24-hour rhythmicity in untrained C57BL/6J control mice (Supplementary Figure 1G; F_(1,17)_ = 14.48, p = 0.0014; amplitude = 23.98, 95% CI 10.68-37.27; R² = 0.460), with low levels during the light phase and a significant increase during the dark phase. Cosinor analysis was also significant in h*SNCA*^A53T^ *Gba1*^+/+^ (Supplementary Figure 1H; F_(1,17)_ = 8.473, p = 0.0097; amplitude = 11.56; 95% CI: 3.18-19.93; R² = 0.333), while it showed not significant rhythmicity in h*SNCA*^A53T^ *Gba1*^+/L444P^ mice (Supplementary Figure 1I; F_(1,17)_ = 3.231, p = 0.0900; amplitude = 6.587; 95% CI: 1.14-14.32; R² = 0.159), however the pattern of GCase activity was markedly altered relative to controls. Following daily rotarod training during the dark (active) phase at ZT16 (*t4*), cosinor analyses were consistent with significant 24-hour rhythmicity in C57BL/6J control mice (Supplementary Figure 1J; F_(1,17)_ = 6.276, p = 0.0227; amplitude = 13.37, 95% CI 2.11-24.63; R² = 0.270). Although the cosinor analyses resulted significant also in both h*SNCA*^A53T^ *Gba1*^+/+^ mice (Supplementary Figure 1K; (F_(1,17)_ = 11.02, p = 0.0040; amplitude = 4.951, 95% CI 1.81–8.10; R² = 0.393) and h*SNCA*^A53T^ *Gba1*^+/L444P^ mice (Supplementary Figure 1L; F_(1,17)_ = 14.42, p = 0.0014; amplitude = 7.578, 95% CI 3.37-11.79; R² = 0.459), nonetheless the pattern of GCase activity was markedly altered relative to controls.

Overall, these cosinor analyses are consistent with the pattern of GCase activity observed across experimental groups and conditions. However, as noted above, they are purely descriptive.

*Please, insert Supplementary Figure 1 about here.*

# Supplementary Figure 1 legend

**Supplementary Figure 1. Cosinor analysis of plasma GCase activity across genotypes, housing conditions, and training status.** Plasma GCase activity was fitted to a 24-hour sine wave by nonlinear regression, and the fit with amplitude unconstrained was compared to a null model with amplitude fixed to zero (extra sum-of-squares F-test) to assess 24-hour rhythmicity. As this approach does not account for the repeated-measures structure of the data, the reported statistics should be interpreted as descriptive indices of goodness-of-fit rather than as formal, independent evidence of rhythmicity. Data points represent individual animals (n = 5/genotype/condiction); curve lines represent the fitted cosinor curve. Individual animal trajectories are overlaid on the group-level fitted curve for illustrative purposes; the statistics reported below are derived from the pooled nonlinear regression described in Methods. (A-C) Standard light/dark cycle (ZT0 = 9AM), untrained. Cosinor analysis did not reach significance in C57BL/6J mice (A; p = 0.2177) but reached significance in both hSNCA^A53T^ Gba1^+/+^ (B; p = 0.0249, *) and h*SNCA*^A53T^ *Gba1*^+/L444P^ mice (C; p = 0.0294, *). (D-F) Standard light/dark cycle, after two weeks of daily rotarod training at ZT4. No significant rhythmicity was detected in C57BL/6J mice (D; p = 0.1306) or in hSNCA^A53T^ Gba1^+/+^ mice (E; p = 0.3163), whereas training strengthened the pattern in h*SNCA*^A53T^ *Gba1*^+/L444P^ (F; p = 0.0013, **). (G-I) Reversed light/dark cycle (ZT0 = 9PM), untrained. Cosinor analysis reached significance in C57BL/6J control mice (G; p = 0.0014, **) and in hSNCA^A53T^ Gba1^+/+^ mice (H; p = 0.0097, **), but not in h*SNCA*^A53T^ *Gba1*^+/L444P^ mice (I; p = 0.0900). (J-L) Reversed light/dark cycle, after daily rotarod training at ZT16. Cosinor analysis reached significance in C57BL/6J control mice (J; p = 0.0227, *), hSNCA^A53T^ Gba1^+/+^ (K; p = 0.0040, **), and h*SNCA*^A53T^ *Gba1*^+/L444P^ mice (L; p = 0.0014, **), although the overall pattern of GCase activity remained markedly altered relative to controls in both PD-mutant genotypes.
